# Performance of USPSTF-recommended osteoporosis risk assessment tools in identifying osteoporosis in older men: a multicentre retrospective study

**DOI:** 10.3389/fendo.2025.1729945

**Published:** 2025-12-15

**Authors:** Xin Li, Dongsheng Guo, Chen Yan, Mingran Luo, Yixin Liang, Jian Zhang, Lin Cheng, Yuefu Dong

**Affiliations:** 1 Department of Orthopedics, The Affiliated Lianyungang Hospital of Xuzhou Medical University (The First People’s Hospital of Lianyungang), Lianyungang, Jiangsu, China; 2 Department of Orthopedics, The Affiliated Hospital of Xuzhou Medical University, Xuzhou, Jiangsu, China; 3 Department of Osteoporosis, The Affiliated Lianyungang Hospital of Xuzhou Medical University (The First People’s Hospital of Lianyungang), Lianyungang, Jiangsu, China

**Keywords:** men, osteoporosis, risk assessment, screening, USPSTF

## Abstract

**Background:**

Despite the growing recognition of osteoporosis in men, screening tools validated for this population remain underutilized. This study aimed to evaluate the diagnostic performance of three U.S. Preventive Services Task Force (USPSTF)–recommended risk assessment tools for identifying osteoporosis among older men.

**Methods:**

This retrospective cross-sectional study included 2,824 men aged ≥65 years who underwent dual-energy X-ray absorptiometry (DXA) at two teaching hospitals between 2015 and 2024. Osteoporosis was defined as a T-score ≤ −2.5 at the femoral neck, total hip, or lumbar spine. Three screening tools, the Osteoporosis Self-Assessment Tool (OST), Osteoporosis Index of Risk (OSIRIS), and Simple Calculated Osteoporosis Risk Estimation (SCORE)—were calculated for each participant. Diagnostic accuracy was assessed using receiver operating characteristic (ROC) curve analysis, with the area under the curve (AUC) compared using the DeLong test. Optimal thresholds were identified by maximizing the Youden index.

**Results:**

Among the 2,824 men included in the study, 598 (21.2%) were diagnosed with osteoporosis. All three USPSTF-recommended tools demonstrated acceptable discrimination. The AUCs were 0.7339 (95% CI, 0.7121–0.7556) for OST, 0.7168 (95% CI, 0.6939–0.7396) for OSIRIS, and 0.7153 (95% CI, 0.6931–0.7375) for SCORE. Pairwise comparisons showed that OST performed significantly better than OSIRIS (ΔAUC = 0.0171, p = 0.0124) and SCORE (ΔAUC = 0.0186, p = 0.0002), whereas OSIRIS and SCORE demonstrated comparable performance (ΔAUC = 0.0015, p = 0.8523). The optimal thresholds were −0.7 for OST, −1.5 for OSIRIS, and 11.5 for SCORE, producing sensitivities of 0.7743, 0.6739, and 0.6890 and specificities of 0.5714, 0.6698, and 0.6352.

**Conclusions:**

Among USPSTF-endorsed screening tools, OST demonstrated the best overall diagnostic performance for identifying osteoporosis in older men. Its simplicity and favorable sensitivity–specificity balance support its utility as a practical first-line screening approach in clinical settings.

## 1 Introduction

Osteoporosis in men is increasingly recognized as a major public health concern but remains substantially underdiagnosed and undertreated (1, 2). Although the lifetime risk of osteoporotic fracture in men is estimated to be up to one in five, clinical screening and preventive strategies continue to focus predominantly on women (3). Fractures in older men, particularly hip fractures, are associated with greater morbidity, prolonged disability, and higher mortality compared with those in women, underscoring the need for early detection and intervention (4). In China, recent multicenter epidemiologic data indicate that the age-standardized prevalence of osteoporosis in middle-aged and older men is approximately 20.73%, underscoring the substantial burden of osteoporosis in this population (5).

Current guidelines by the U.S. Preventive Services Task Force (USPSTF) recommend routine osteoporosis screening for women aged 65 years and older but state that evidence is insufficient to support screening in men (6). As a result, most older men undergo bone mineral density (BMD) testing only after sustaining a fracture, despite accumulating evidence that age- and weight-based clinical indices may help identify those at high risk (7–10).

Among existing clinical screening tools, the Osteoporosis Self-Assessment Tool (OST) (7, 11, 12), the Osteoporosis Index of Risk (OSIRIS) (13–15), and the Simple Calculated Osteoporosis Risk Estimation (SCORE) (16–18) are the three instruments endorsed by the USPSTF for pre-screening decisions before dual-energy X-ray absorptiometry (DXA). These tools were originally developed and validated primarily in postmenopausal women, with limited and inconsistent evidence regarding their diagnostic accuracy in older men (19, 20). Given sex-specific differences in body composition, fracture patterns, and hormonal influences on bone metabolism, their performance in male populations cannot be assumed to mirror that observed in women (2, 21, 22). Given that fracture risk and bone loss accelerate notably after age 65 in men, this age group represents a critical window for early identification and intervention (23).

Despite the growing clinical importance of male osteoporosis, data on the comparative diagnostic performance of these USPSTF-recommended risk assessment tools in men aged 65 years or older remain scarce. This knowledge gap limits the establishment of efficient case-finding strategies for this under-screened yet high-risk population.

Therefore, the present study aimed to evaluate and compare the diagnostic performance of three widely used, USPSTF-endorsed osteoporosis risk assessment tools—OST, OSIRIS, and SCORE—for identifying DXA-defined osteoporosis in men aged 65 years and older.

## 2 Materials and methods

### 2.1 Study design

This retrospective cross-sectional study was conducted at two teaching hospitals between January 2015 and December 2024. The study protocol was reviewed and approved by the institutional ethics committees of both hospitals. Because of the retrospective design, the requirement for informed consent was waived. All procedures were performed in accordance with the ethical standards of the Declaration of Helsinki.

### 2.2 Participants

Men aged 65 years or older who underwent DXA during the study period were included.

**Figure 1:**
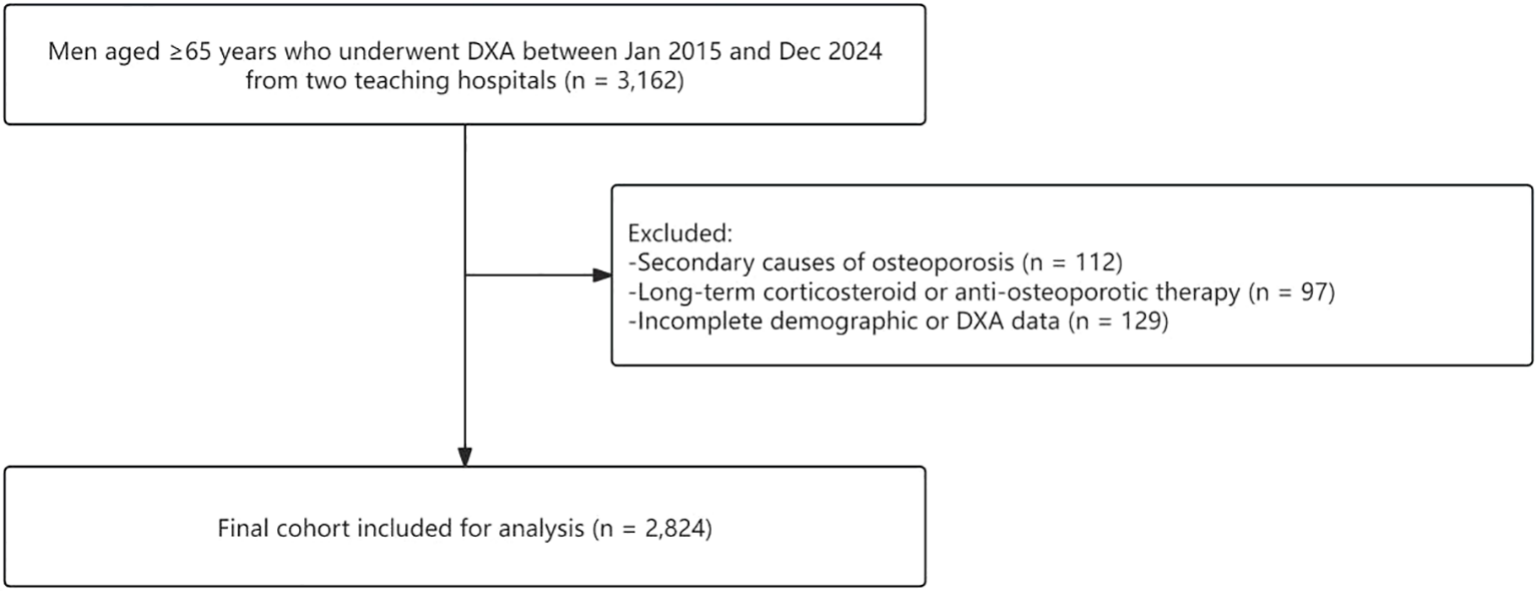
Flowchart showing participant selection for analysis.

Exclusion criteria were: (1) secondary causes of osteoporosis, such as endocrine or metabolic disorders, malignancy, or chronic kidney disease; (2) long-term corticosteroid therapy or use of medications known to affect bone metabolism; and (3) incomplete demographic or DXA data. The detailed participant selection process is illustrated in **Figure 1**.

DXA testing was ordered by physicians according to their clinical judgement and local practice rather than by a standardized prescreening algorithm or formal risk assessment tool. No additional prescreening criteria beyond age and clinical indications were used to determine eligibility for DXA.

### 2.3 Data collection

Demographic and clinical data were extracted from the electronic medical record systems of the two hospitals. Variables included age, weight, height, body mass index (BMI), smoking and alcohol consumption, comorbidities (hypertension, diabetes mellitus, and coronary artery disease), and history of low-trauma fracture.

Bone mineral density (BMD) was measured at the lumbar spine (L1–L4), femoral neck, and total hip using dual-energy X-ray absorptiometry (DXA; Hologic Discovery Series, Hologic Inc., Marlborough, MA, USA). The instruments were calibrated daily according to the manufacturer’s quality-control procedures.

According to the World Health Organization (WHO) diagnostic criteria, osteoporosis was defined as a T-score of −2.5 or lower at any of these sites. T-scores were derived from the National Health and Nutrition Examination Survey (NHANES) phase III reference database, consistent with the normative reference used in the Hologic system and recommended by the 2007 WHO technical report.

### 2.4 Risk assessment tools

Three risk assessment tools recommended by the USPSTF were evaluated: OST, OSIRIS, and SCORE (24–26).

Scores were calculated using the following published formulas:


$$\begin{array}{l} \text{OST}=(\text{weight }[\text{kg}]-\text{age[years]})\times 0.2\\ \text{OSIRIS}=-\text{round}(\text{Age}/5)+\text{round}(\text{Weight}/5)+2\\ \times (\text{Estrogen use})-2\times (\text{Prior fracture})\\ \text{SCORE}=\text{weighted score derived from race, rheumatoid arthritis,}\\ \text{fracture history, estrogen use, age, and weight} \end{array}$$

For OST and OSIRIS, body weight was entered in kilograms, consistent with the measurements obtained during the DXA visit. For SCORE, weight in kilograms was converted to pounds in accordance with the original SCORE equation. In this male cohort, the “estrogen use” component of the SCORE equation was uniformly coded as 0, consistent with prior validation studies in men. Lower OST values, lower OSIRIS values, and higher SCORE values indicate a higher risk of osteoporosis.

### 2.5 Statistical analysis

Continuous variables were summarized as median with interquartile range (IQR), and categorical variables were presented as counts and percentages (n, %). Between-group comparisons were performed using the Mann–Whitney U test for continuous variables and the chi-square or Fisher’s exact test for categorical variables as appropriate. Baseline characteristics were summarized for the overall sample and according to osteoporosis status.

Sample size justification was based on a precision-based approach using the Hanley–McNeil variance method for ROC curves implemented in R. We required the 95% confidence interval half-width of the AUC estimate to be no greater than 0.04. Assuming an expected AUC of 0.70 and a disease prevalence of 20%, the Hanley–McNeil formula indicated that approximately 1,185 participants (237 osteoporotic cases and 948 non-osteoporotic controls) would be required to achieve this level of precision. Our final study cohort of 2,824 men therefore exceeds the required sample size by more than a factor of two, ensuring excellent statistical precision for the ROC analyses and pairwise DeLong comparisons.

The diagnostic performance of each screening tool for identifying osteoporosis was evaluated using receiver operating characteristic (ROC) curve analysis. The area under the ROC curve (AUC) and its 95% confidence interval (CI) were calculated using the DeLong method. Optimal cut-off values for each tool were determined by maximizing the Youden index (sensitivity + specificity − 1). Sensitivity, specificity, positive predictive value (PPV), and negative predictive value (NPV) were calculated at these thresholds to quantify diagnostic accuracy. Bootstrap resampling with 2,000 iterations was used to estimate 95% CIs for sensitivity and specificity at the optimal cut-offs. Pairwise differences in AUCs were compared using the DeLong test for correlated ROC curves. All analyses were conducted using R software (version 4.3.1). A two-sided p value<0.05 was considered statistically significant.

## 3 Results

### 3.1 Baseline characteristics

A total of 2,824 men aged ≥65 years who underwent DXA were included, of whom 598 (21.2%) were diagnosed with osteoporosis based on a T-score ≤ −2.5 at the femoral neck, total hip, or lumbar spine.

As shown in **Table 1**, men with osteoporosis were significantly older than those without osteoporosis (76 [71–80] *vs*. 72 [68–76] years, p< 0.001). They also had lower body weight (65 [59–71] *vs*. 71 [65–77] kg, p< 0.001) and BMI (22.7 [20.4–25.2] *vs*. 24.7 [22.5–27.4] kg/m², p< 0.001). BMD values at the femoral neck, total hip, and lumbar spine were substantially lower in the osteoporosis group (all p< 0.001).

**Table 1:** Baseline characteristics of the study population according to osteoporosis status.

Variable	Non-osteoporosis (n=2226)	Osteoporosis (n=598)	P value
Age	72 (68 - 76)	76 (71 - 80)	<0.001
Height cm	169 (164 - 173)	169 (165 - 173)	0.704
Weight kg	71 (65 - 77)	65 (59 - 71)	<0.001
BMI	24.7 (22.5 - 27.4)	22.7 (20.4 - 25.2)	<0.001
BMD FemoralNeck	−1.39 (−1.85 - −0.90)	−2.71 (−3.07 - −2.52)	<0.001
BMD TotalHip	−1.44 (−1.82 - −0.99)	−2.57 (−2.84 - −2.33)	<0.001
BMD LumbarSpine	−1.45 (−1.83 - −0.99)	−2.57 (−2.82 - −2.30)	<0.001
Smoking Current			0.312
No	1637 (73.5%)	452 (75.6%)	
Yes	589 (26.5%)	146 (24.4%)	
Alcohol Use			0.196
No	1492 (67.0%)	384 (64.2%)	
Yes	734 (33.0%)	214 (35.8%)	
Hypertension			0.844
No	1062 (47.7%)	288 (48.2%)	
Yes	1164 (52.3%)	310 (51.8%)	
Diabetes			0.108
No	1765 (79.3%)	456 (76.3%)	
Yes	461 (20.7%)	142 (23.7%)	
Coronary Artery Disease			0.177
No	1868 (83.9%)	488 (81.6%)	
Yes	358 (16.1%)	110 (18.4%)	
Prior LowTrauma Fracture			<0.001
No	2010 (90.3%)	500 (83.6%)	
Yes	216 (9.7%)	98 (16.4%)	
OST	−0.2 [−1.8 - 1.0]	−2.2 [−3.6 - −0.8]	<0.001
OSIRIS	0 [-2 - 1]	-2 [-4 - -1]	<0.001
SCORE Rheumatoid Arthritis			0.981
No	2074 (93.2%)	557 (93.1%)	
Yes	152 (6.8%)	41 (6.9%)	
SCORE Prior Fracture			<0.001
No	2010 (90.3%)	500 (83.6%)	
Yes	216 (9.7%)	98 (16.4%)	
SCORE Estrogen Use			
No	2226 (100.0%)	598 (100.0%)	
Yes	0 (0%)	0 (0%)	
CORE Total	10.00 (8.00, 12.00)	13.00 (11.00, 14.00)	<0.001

The distributions of smoking status, alcohol use, hypertension, diabetes, and coronary artery disease did not differ significantly between groups. However, prior low-trauma fracture was more common in men with osteoporosis (16.4% *vs*. 9.7%, p< 0.001).

Risk assessment scores also differed between groups. Participants with osteoporosis had lower OST and OSIRIS values, but higher SCORE values compared with those without osteoporosis (all p< 0.001).

### 3.2 Diagnostic performance of screening tools

**Figure 2:**
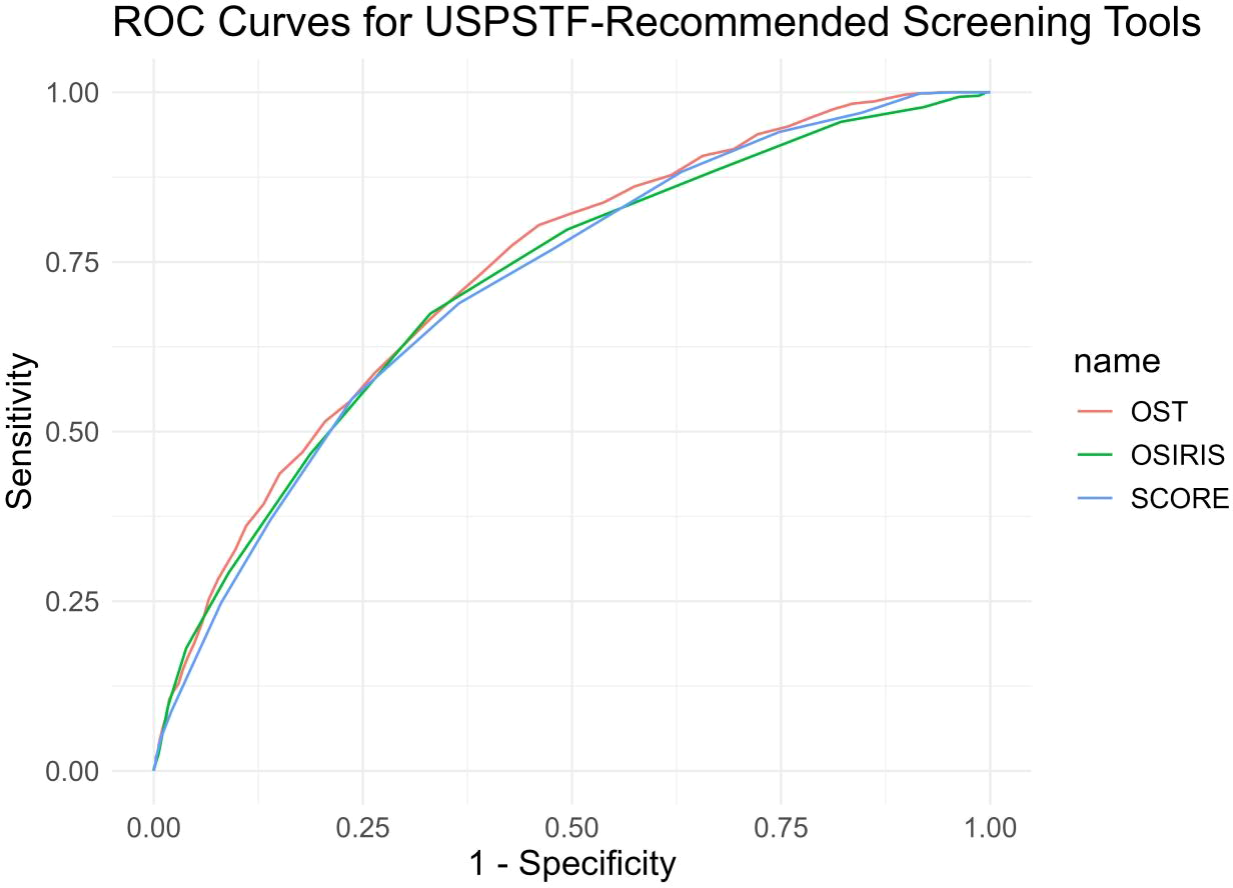
Receiver operating characteristic (ROC) curves of the three USPSTF-recommended screening tools for detecting osteoporosis in older men.

The diagnostic performance of OST, OSIRIS, and SCORE is summarized in **Table 2** and illustrated in **Figure 2**. All three USPSTF-recommended screening tools demonstrated acceptable discrimination for detecting DXA-defined osteoporosis in men aged 65 years or older. Based on the most recent analysis, OST achieved the highest area under the ROC curve, with an AUC of 0.7339 (95% CI, 0.7121–0.7556), followed by OSIRIS with an AUC of 0.7168 (95% CI, 0.6939–0.7396) and SCORE with an AUC of 0.7153 (95% CI, 0.6931–0.7375).

**Table 2:** Diagnostic performance of USPSTF-recommended screening tools for identifying osteoporosis in men aged ≥65 years.

Tool	AUC (95% CI)	ΔAUC *vs* Comparator	Z	p-value	95% CI for ΔAUC
OST	0.7339 (0.7121–0.7556)	*vs* OSIRIS: 0.0171	2.5010	0.0124	0.0037–0.0305
OST	0.7339 (0.7121–0.7556)	*vs* SCORE: 0.0186	3.6815	0.0002	0.0087–0.0285
OSIRIS	0.7168 (0.6939–0.7396)	*vs* SCORE: 0.0015	0.1862	0.8523	−0.0139–0.0169
SCORE	0.7153 (0.6931–0.7375)	—	—	—	—

AUC, area under the receiver operating characteristic curve. DeLong’s test was used for pairwise comparisons of correlated ROC curves. Z, test statistic from DeLong’s test for pairwise comparison of correlated ROC curves.

Pairwise DeLong comparisons showed that OST performed significantly better than OSIRIS (ΔAUC = 0.0171, Z = 2.5010, p = 0.0124) and demonstrated a further improvement over SCORE (ΔAUC = 0.0186, Z = 3.6815, p = 0.0002). OSIRIS and SCORE did not differ significantly (ΔAUC = 0.0015, Z = 0.1862, p = 0.8523). Although the performance advantage of OST over OSIRIS and SCORE was statistically significant, the magnitude of these differences remained modest. Overall, OST showed the strongest discriminative ability among the three tools, while OSIRIS and SCORE exhibited comparable performance patterns.

Visual evaluation of the ROC curves confirmed these findings, with the OST curve demonstrating consistently superior separation between osteoporotic and non-osteoporotic individuals across the range of decision thresholds.

### 3.3 Optimal cut-off values and diagnostic indices

Optimal thresholds were determined using the Youden index and are presented in **Table 3**. The optimal cut-off for OST was −0.7, resulting in a sensitivity of 0.7743 (95% CI, 0.7391–0.8077) and a specificity of 0.5714 (95% CI, 0.5512–0.5912). OSIRIS demonstrated a balanced pattern at its optimal threshold of −1.5, yielding a sensitivity of 0.6739 (95% CI, 0.6371–0.7107) and a specificity of 0.6698 (95% CI, 0.6491–0.6887). SCORE, tested at an optimal cut-off of 11.5, achieved a sensitivity of 0.6890 (95% CI, 0.6505–0.7224) and a specificity of 0.6352 (95% CI, 0.6159–0.6545). The Youden indices were similar across the three tools, measured as 0.3461 for OST, 0.3433 for OSIRIS, and 0.3242 for SCORE.

**Table 3:** Optimal cut-off values and diagnostic indices for the three screening tools.

Tool	Optimal cut-off	Sensitivity (95% CI)	Specificity (95% CI)	Youden index	PPV (%)	NPV (%)
OST	−0.7	0.7743 (0.7391–0.8077)	0.5714 (0.5512–0.5912)	0.3461	9.5881	67.3023
OSIRIS	−1.5	0.6739 (0.6371–0.7107)	0.6698 (0.6491–0.6887)	0.3433	11.5727	64.6181
SCORE	11.5	0.6890 (0.6505–0.7224)	0.6352 (0.6159–0.6545)	0.3242	11.625	66.3399

PPV, positive predictive value; NPV, negative predictive value. Cut-offs were determined by maximizing the Youden index.

With respect to predictive values, OST produced a PPV of 9.5881% and an NPV of 67.3023%, while OSIRIS produced a PPV of 11.5727% and an NPV of 64.6181%. SCORE showed a PPV of 11.625% and an NPV of 66.3399%. These values reflected the moderate prevalence of osteoporosis within the study cohort and reinforced the utility of these tools as prescreening rather than diagnostic instruments.

**Figure 3:**
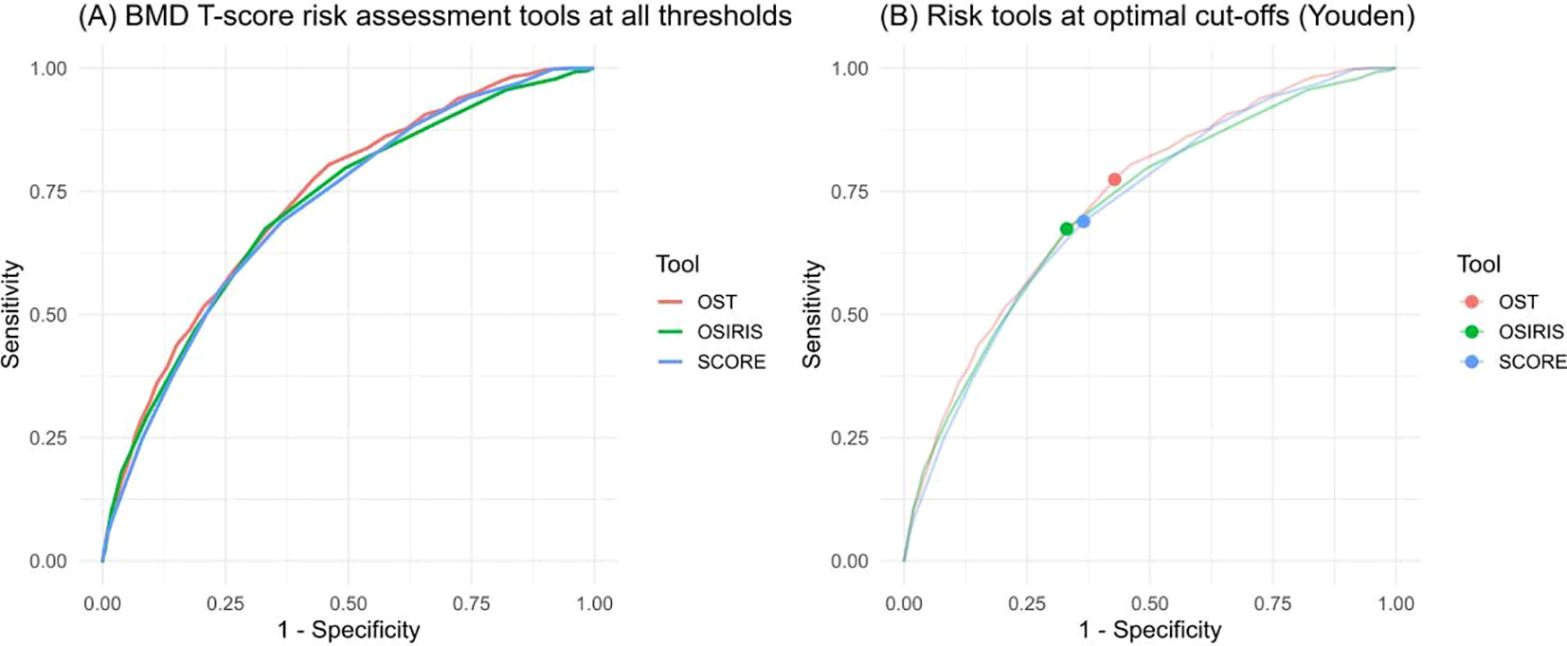
ROC curves at all thresholds **(A)** and at the optimal cut-offs **(B)** based on the Youden index.

Inspection of threshold-specific ROC curves (**Figure 3**) further supported these results. OST demonstrated superior sensitivity while maintaining moderate specificity, whereas OSIRIS and SCORE offered slightly more balanced accuracy profiles. Overall, OST provided the best single-threshold performance for identifying men who would benefit from follow-up DXA testing.

## 4 Discussion

In this two-center retrospective study of 2,824 men aged 65 years and older, three widely used osteoporosis screening tools recommended by the USPSTF—OST, OSIRIS, and SCORE—were systematically evaluated and compared. All three indices demonstrated acceptable discrimination for identifying DXA-defined osteoporosis, with AUC values of 0.7339 for OST, 0.7168 for OSIRIS, and 0.7153 for SCORE. Among them, OST achieved the highest overall discriminative ability, with a statistically significant but small advantage over OSIRIS and a modest advantage over SCORE. At the optimal thresholds determined by the Youden index (−0.7 for OST, −1.5 for OSIRIS, and 11.5 for SCORE), the OST yielded the highest Youden index, reflecting the best overall diagnostic trade-off at its optimal cut-off, confirming its suitability as a simple and practical prescreening tool for older men.

The present results extend previous work that has predominantly focused on postmenopausal women. Earlier studies in female populations reported AUCs typically ranging from 0.60 to 0.70 for OST, OSIRIS, and ORAI or SCORE, indicating modest diagnostic utility (14). In a recent analysis, which included 6,067 postmenopausal women aged 50–64 years, the AUCs were 0.654 for OST, 0.633 for OSIRIS, and 0.663 for ORAI, suggesting limited discrimination even after optimizing cut-off points by the Youden index (25). In contrast, the present study demonstrated notably higher AUCs for all three tools, representing an approximately 0.08 absolute improvement over female cohorts.

This difference likely reflects the higher baseline prevalence of osteoporosis in older men and the greater predictive contrast between weight and age in the elderly male population (2, 27, 28). Unlike women, whose bone loss accelerates during menopause but later plateaus, men experience a more gradual but persistent decline in bone mass and lean body composition, which may make anthropometric indices such as OST more sensitive (29–31). Additionally, the inclusion of weight and age as core components in all three models aligns well with the risk profile of older men, for whom these variables strongly correlate with BMD. The findings also align with recent Asian and European studies, in which OST or OSTA consistently achieved AUCs around 0.70–0.80 for identifying individuals at risk of osteoporosis, performing comparably or better than more complex multivariable models requiring biochemical inputs (32–34).

Our findings are broadly consistent with studies that have evaluated bone screening strategies in men. In a community-based cohort of Chinese men, three simple tools based on age, weight and prior fracture history showed AUCs around 0.80 for DXA-defined osteoporosis and reduced the number of DXA examinations while missing only a small proportion of cases, illustrating the ability of parsimonious indices to enrich high-risk male populations for further testing (7). Similarly, in a Taiwanese study of men aged 50 years and older, the MOSTAi self-assessment tool, which also relies on age and body weight, achieved an AUC near 0.70 and was proposed as a first-line screener (35). More advanced approaches have focused on refining risk stratification rather than replacing simple tools. The STRAMBO study in European men showed that a finite-element–derived bone strain index from hip DXA scans predicted incident fractures independently of BMD and FRAX, suggesting that image-derived mechanical metrics can add prognostic information once men have been selected for densitometry (36). A cluster randomized trial in primary care demonstrated that electronic health record–based case finding combined with a bone health service increased DXA testing and osteoporosis treatment in men at risk (1). Together, these data support our conclusion that simple age–weight–based tools are useful front-end filters in men, but optimal fracture prevention will require integration with more sophisticated imaging and system-level interventions.

The current findings have important implications for clinical practice. In real-world settings, the decision to perform DXA testing in men is often subjective, limited by resource availability, and heavily influenced by visible comorbidities rather than systematic risk assessment (21, 37). Implementing simple screening tools such as OST can improve efficiency by identifying individuals most likely to benefit from confirmatory BMD measurement. The OST formula requires no laboratory tests and can be applied instantly at the bedside, in outpatient clinics, or even via community health questionnaires.

At the optimal threshold (OST< −0.7 in this cohort), the sensitivity of approximately 77% suggests that more than three-quarters of men with osteoporosis could be correctly flagged for further DXA screening, while maintaining moderate specificity to avoid unnecessary testing. Given the high morbidity and mortality associated with osteoporotic fractures in men, particularly hip fractures, the potential population-level impact of adopting such low-cost tools in primary care and rehabilitation settings is substantial (38–40). The present findings therefore support expanding USPSTF-style prescreening frameworks to male populations, where structured risk assessment remains underutilized.

This study has several strengths. It represents one of the largest male cohorts to systematically compare the three USPSTF-endorsed screening tools using standardized DXA data. The inclusion of men exclusively aged ≥65 years ensures homogeneity in age-related bone loss and directly addresses the current evidence gap in this clinically relevant group. The use of bootstrapped confidence intervals and DeLong tests provided robust estimates of model performance, and the multi-site data source enhanced generalizability.

However, several limitations should be acknowledged. First, the retrospective cross-sectional design precludes causal inference and limits assessment of longitudinal fracture prediction. Second, although the cohort size was large, it was derived from two teaching hospitals, which may not fully represent the general male population. Third, the performance of OSIRIS and SCORE were somewhat constrained by the exclusion of the estrogen-use variable, which is uniformly coded as zero in men, slightly reducing its theoretical variance. Finally, while Youden index optimization identified ideal thresholds for this population, external validation in prospective, multi-ethnic cohorts remains necessary before clinical implementation.

## 5 Conclusions

All three USPSTF-recommended screening tools—OST, OSIRIS, and SCORE—showed acceptable diagnostic performance for identifying osteoporosis in older men. Among them, OST demonstrated the highest AUC and the best diagnostic performance at its optimal threshold, supporting its use as a simple, inexpensive, and practical prescreening tool to guide DXA testing and facilitate earlier detection and management of osteoporosis in men.

## Data Availability

The raw data supporting the conclusions of this article will be made available by the authors, without undue reservation.
